# Neuromuscular Junction Protection for the Potential Treatment of Amyotrophic Lateral Sclerosis

**DOI:** 10.1155/2012/379657

**Published:** 2012-08-07

**Authors:** Dan Krakora, Corey Macrander, Masatoshi Suzuki

**Affiliations:** ^1^Department of Comparative Biosciences, University of Wisconsin-Madison, 2015 Linden Drive, Madison, WI 53706, USA; ^2^The Stem Cell and Regenerative Medicine Center, University of Wisconsin-Madison, Madison, WI 53705, USA

## Abstract

Amyotrophic lateral sclerosis (ALS) is a neuromuscular disease characterized by the progressive degeneration of upper and lower motor neurons (MNs), leading to muscular atrophy and eventual respiratory failure. ALS research has primarily focused on mechanisms regarding MN cell death; however, degenerative processes in the skeletal muscle, particularly involving neuromuscular junctions (NMJs), are observed in the early stages of and throughout disease progression. According to the “dying-back” hypothesis, NMJ degeneration may not only precede, but actively cause upper and lower MN loss. The importance of NMJ pathology has relatively received little attention in ALS, possibly because compensatory mechanisms mask NMJ loss for prolonged periods. Many mechanisms explaining NMJ degeneration have been proposed such as the disruption of anterograde/retrograde axonal transport, irregular cellular metabolism, and changes in muscle gene and protein expression. Neurotrophic factors, which are known to have neuroprotective and regenerative properties, have been intensely investigated for their therapeutic potential in both the preclinical and clinical setting. Additional research should focus on the potential of preserving NMJs in order to delay or prevent disease progression

## 1. Introduction

**Figure 1:**
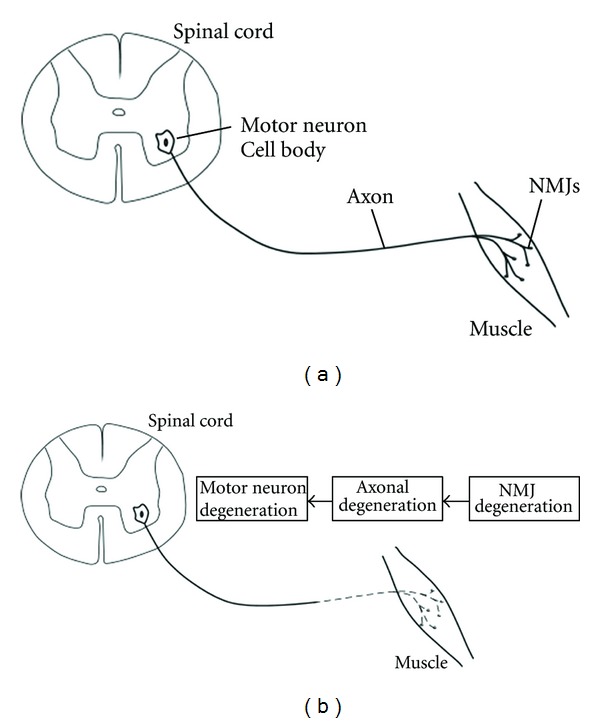
Schematic illustrating the “Dying-Back” hypothesis. (a) In a healthy system, communication and the transport of vital biomolecules occurs normally along the axon connecting MNs and the NMJs they innervate. (b) In ALS, a progressive distal to proximal degeneration occurs, described as “Dying-Back.” NMJ degeneration is followed by axonal degeneration and eventually MN degeneration.

Amyotrophic Lateral Sclerosis (ALS) is a fatal neurodegenerative disease characterized by the loss of both upper and lower motor neurons (MNs) [1–3]. ALS research has primarily focused on mechanisms regarding MN cell death; however, degenerative processes in the skeletal muscle, particularly involving neuromuscular junctions (NMJs), are observed in the early stages of and throughout disease progression [4, 5]. Many studies support a “dying-back” hypothesis in which distal NMJ degeneration precedes and causes proximal cell body death. This paper will describe the NMJ, ALS pathology and the “dying-back” hypothesis (Figure 1). Then, we will discuss growth factor treatments and current progress regarding them.

## 2. Amyotrophic Lateral Sclerosis

Amyotrophic lateral sclerosis (ALS) is a neuromuscular disease characterized by the progressive degeneration of upper and lower MNs, leading to muscular atrophy and eventual respiratory failure [1–3]. Diagnoses occur most often between the ages of 40 and 60 and the disease is fatal within 5-6 years of clinical diagnosis. ALS is the most frequent adult-onset MN disease with a worldwide incidence rate of 1–3 new cases per 100,000 individuals. About 90% of ALS cases are sporadic and the remaining 10% of ALS cases are familial (FALS). In about 20% of FALS cases, the cause can be attributed to a mutation in the Cu^2+/^Zn^2+^ superoxide dismutase 1 (SOD1), a ubiquitously-expressed free-radical defense enzyme [6]. The mutations cause misfolding of this normally stable homodimeric protein [7]. Overexpressing the human SOD1 mutant in rodents results in a disease progression similar to that observed in ALS patients, providing a valuable model (SOD1^G93A^ mice or rats) on which a great deal of ALS research has been based [8, 9]. Two other heritable mutations associated with protein mislocalization and aggregation have become important areas of research in ALS: the RNA-processing proteins fused in sarcoma (FUS) and TARDNA binding protein 43 (TDP-43) [10, 11]. Although the SOD1 mutation represents a relatively rare, inherited form of ALS, both inherited and sporadic forms of ALS exhibit the same clinical course and neuropathology. Therefore, SOD1^G93A^ rodent models are important tools with which to better understand and investigate potential therapeutic treatments for ALS.

The mechanism underlying MN death in ALS is still unknown. Multiple mechanisms account for the selective vulnerability of MNs, including abnormal astrocyte and microglial activation, reduced neurotrophic factor secretion, protein aggregations, mitochondrial malfunction, rupture in the axonal passage, destruction in calcium metabolism, changes in skeletal proteins, high levels of excitotoxicity by glutamate and oxidative damage [12–14]. It is widely accepted that ALS is caused by MN degeneration. However, NMJ degeneration precedes and may even directly cause MN loss.

## 3. The Neuromuscular Junction

**Figure 2:**
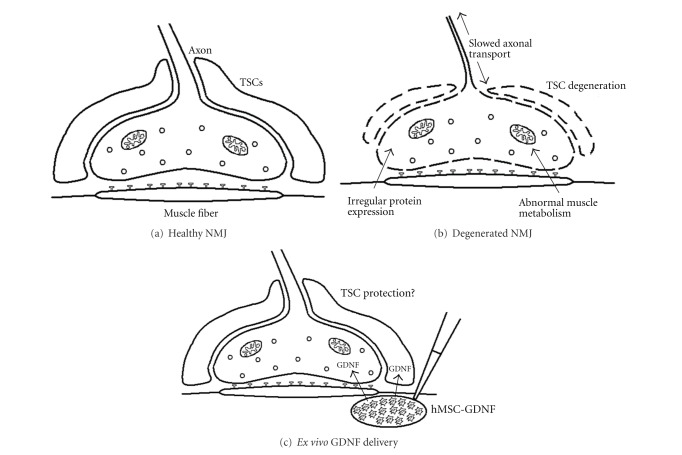
Schematic detailing NMJ degeneration and treatment. (a) A healthy, functioning NMJ, including TSCs and Ach receptors, is a vital point of communication between MNs in the spinal cord and muscle. (b) In ALS, NMJs begin to degenerate due to a number of pathologies, including disrupted axonal transport and irregular mitochondrial metabolism. NMJ degeneration occurs long before MN degeneration in the spinal cord, preceding clinical symptoms. (c) *Ex vivo* delivery of GDNF to NMJ via hMSC-GDNF may help to rescue TSCs from degenerative processes, thereby, delaying or preventing degeneration of the NMJ as a whole.

The neuromuscular junction (NMJ) is the synapse where the axon terminal of a MN meets the motor endplate, the highly excitable region of muscle fiber plasma membrane responsible for initiating action potentials across the muscle's surface, ultimately causing the muscle to contract. (Figure 2(a)). In vertebrates, the signal passes through the NMJ via the neurotransmitter acetylcholine. Terminal branches expand outward from the motor nerve and emerge from their myelin sheath at the muscle to form terminals. These terminals are filled with synaptic vesicles, mitochondria, and tubules from smooth endoplasmic reticula. Synaptic terminals permit the necessary communication between MNs and their target muscles for muscle contraction. The motor end plate is densely populated by nicotinic acetylcholine receptors. Glial cells, called terminal Schwann cells (TSCs), are also intimately associated with the nerve-muscle connection. TSCs are nonmyelinating Schwann cells that play important roles in the formation, function, maintenance, and repair of the NMJ [15]. In neuromuscular junction diseases such as myasthenia gravis, Lambert-Eaton syndrome, and myasthenic syndrome, normal conduction through the neuromuscular junction is disrupted [16]. 

## 4. Presymptomatic Degeneration of the NMJ

 Recent studies suggest that distal degeneration in the skeletal muscle plays a key role in the progression of ALS. Several studies using SOD1^G93A^ mice have shown that NMJ degeneration occurs in the early stages of disease progression, long before MN loss [17]. Furthermore, distal axonopathy followed NMJ denervation, but preceded both neuronal degeneration and the onset of clinical symptoms (Figure 1) [18–21]. There is growing evidence suggesting that muscle weakness is not apparent until a large proportion of the motor units are lost [5]. The time differential between NMJ degeneration and muscle weakness is caused by remaining axonal reinnervation of the muscle. This process is able to compensate for denervation at first and no loss in muscle strength is observed. Eventually, reinnervation is not able to keep up with degeneration from the disease and muscle weakness becomes apparent [14, 15].

Presymtomatic NMJ degeneration is supported by a study that used longitudinal magnetic resonance imaging (MRI) of the same SOD1^G93A^ mice. Researchers discovered that the muscle volume in these animals was significantly reduced from as early as week 8 of life, 4 weeks prior to clinical onset [22]. Neuropathological analysis using SOD1^G93A^ mouse samples demonstrated a similar pattern of disease with prominent evidence of axonal degeneration only in muscle [23]. Furthermore, Hegedus et al. (2007) [24] applied electromyography to the SOD1^G93A^ mice and explored the time course of functional loss in motor units. They also explored whether or not a difference existed between the loss of function in fast and slow twitch muscle. A significant decline in the whole muscle contractile force occurred 50 days before the onset of clinical symptoms. Furthermore, the number of functional motor units decreased in fast twitch, but not slow twitch, muscle. Another study found that skeletal muscle-restricted expression of the mutant SOD1 gene is sufficient to dismantle neuromuscular connections and cause MN distal axonopathy, resulting in MN disease in these mice [25–27]. This suggests that subclinical pathology in skeletal muscle is not merely the consequence of neurogenic atrophy, but initiates additional pathogenic processes. 

The “dying-back” hypothesis is further supported by our recent research using SOD1^G93A^ rats. We assessed whether human neural progenitor cells secreting glial cell line-derived neurotrophic factor (hNPC-GDNF) could also maintain neuromuscular connections following transplantation into the spinal cord of ALS rats. The animals were unilaterally transplanted at presymptomatic 70 days with hNPC-GDNF and then sacrificed at the mid-stage of disease (6 weeks after surgery). We confirmed a highly significant increase in MN survival within the hNPC-GDNF group when compared with the non-grafted side. However, hNPC-GDNF did not have a significant effect on the innervation of NMJs in the hind limb muscle [28]. These results suggest that while hNPC releasing GDNF were able to protect MNs, they were no longer connected to the muscle. 

## 5. Possible Mechanisms of NMJ Denervation

In the last few decades, many explanations regarding NMJ degeneration have been proposed. Here, we describe some of the main components of the “dying-back” hypothesis which have been demonstrated (Figure 2). 

One circumstance that may contribute to NMJ degeneration is the accumulation of SOD1^G93A^ proteins in neurons, which slows anterograde and retrograde axonal transport, resulting in insufficient maintenance of the distal axon [29, 30]. An increased expression of Sema3A, an axon guidance protein, was found in SOD1^G93A^ mice. It is thought that Sema3A, secreted by the TSCs, may lead to the repulsion of motor axons away from the NMJ, resulting in denervation [5]. Nogo-A, a neurite outgrowth inhibitor that is overexpressed in the slow-twitch fibers of SOD1^G93A^ mice, may contribute to NMJ degeneration [31]. Overexpression of the dynamitin subunit of dynactin also inhibits retrograde transport and causes an *α*-MN degeneration progression similar to that observed in ALS [32]. Angiogenin (ANG), which is mainly implicated in angiogenesis, also has axonal guidance functions by regulating neurite extension and pathfinding. Mutations in ANG can inhibit neurite outgrowth and negatively affects MN survival [5]. The consequences of these mutations highlight the importance of anterograde and retrograde transport in maintaining the functionality of MNs. Since the fine-tuning of axonal transport is crucial for the survival of motor neurons, the development of molecular-targeted therapies to maintain axonal transport would be a powerful strategy. 

Recent work has also shown that abnormalities in muscle energy metabolism may play a role in initiating NMJ degeneration. Large MNs are susceptible in a caliber-specific order with the largest caliber axons being the most susceptible to degeneration in SOD1^G93A^ mice and human patients [33, 34]. This is supported by the observation that MNs innervating fast-twitch muscle fibers, mainly composed of type IIB and IID/X muscle fibers, showed signs of degradation before MNs innervating slow-twitch type I and IIA fibers in SOD1^G93A^ rodents. Fast-twitch fibers are often innervated by the larger caliber type II MNs and slow-twitch fibers are innervated by the smaller caliber type I MNs [24]. It is generally accepted that the MNs innervating fast motor units have the largest soma sizes, axon calibers, and innervation ratios [35]. It has been proposed that irregular muscle metabolism is the cause of caliber-specific degradation. SOD1^G93A^ rodents experience an increased basal metabolic rate and subsequent weight loss due to decreased levels of cellular adenosine-5′-triphosphate (ATP) [36]. An increased basal metabolic rate in SOD1^G93A^ rats has also been linked to elevated levels of mitochondrial uncoupling protein [37]. Since larger-caliber nerve fibers have the highest metabolic needs, they would be the most susceptible in irregular metabolic conditions [5]. 

Alterations in trophic factor expression in the skeletal muscle could influence the course of MN degeneration and NMJ denervation. Numerous studies support this idea and demonstrate that the expression of growth factors dramatically changes in the muscle of patients with ALS throughout the stages of the disease. Although increased GDNF mRNA expression was observed in muscle biopsies from ALS patients [38], the other study showed that GDNF mRNA was decreased in the postmortem muscles of ALS patients [39]. These observations imply that GDNF gene expression decreases considerably as the disease progresses. Similarly, decreased expression of insulin-like-growth factor-I (IGF-I) has been observed in the skeletal muscle of ALS patients [40]. 

Furthermore, TSCs may be intimately involved in the course of ALS pathology. TSCs cap the nerve terminal covering motor terminal branches and synaptic boutons. These cells play key roles in the maintenance of preterminal axon structure and function during development and in adult life [41]. TSCs dysfunction or loss could thus serve as a possible trigger for NMJ degeneration. We recently performed a longitudinal study using SOD1^G93A^ rats to understand the ability of TSCs to protect neuromuscular connections and found that the number of TSCs was significantly reduced following disease progression in ALS rat muscle. Given the importance of TSCs in the maintenance and function of NMJs, further studies are necessary to understand the mediators of TSC plasticity. Then, suitable cellular and molecular targets can be identified for novel treatments for ALS and other neuromuscular diseases. 

## 6. Upper MNs and “Dying-Back”

The connection between upper MN degeneration and the dying-back hypothesis is still uncertain. Some early studies suggest that cortical and lower MN degeneration occur independently and not as a transsynaptic phenomenon [42, 43]. Attarian et al. conducted two studies comparing the responses of motor units in ALS patients to transcranial magnetic stimulation and peripheral nerve stimulation [44, 45]. Although a positive correlation existed between cortical and spinal dysfunction at first, it eventually disappeared, again suggesting that upper and lower MN degeneration occur separately. Furthermore, it has been suggested that the disease starts at a focal point which involves both upper and lower MNs, but that each set of MNs is affected separately as the disease progresses [46]. Some studies even suggest a “dying-forward” hypothesis which places corticomotoneuron degeneration at the earlier stages of disease progression. Corticomotoneuron hyperexcitability, induced by glutamate, may drive the anterior horn cell into a metabolic deficit [47]. However, identifying corticospinal MN degeneration and corresponding subcerebral projection neurons more accurately can now be done with recently identified molecular markers and FluoroGold labeling. Only ~6,000 corticospinal and corticobulbar MNs exist per hemisphere in mice, intermixed with millions of other cortical pyramidal neurons in the same region and layer V of the motor cortex [48]. As the pathology and progression of upper and lower MN degeneration is better understood, we can refine our treatment target and rationale.

## 7. Possible Treatments Targeting Muscle: How Can We Prevent “Dying-Back”?

Mounting evidence for the “dying-back” hypothesis suggests that the survival of NMJs is imperative in hindering the progression of ALS. Therefore, therapeutic treatments aimed at preserving NMJs may be the most effective. 

One therapeutic strategy following this model is the direct delivery of neurotrophic factors to skeletal muscle. Neurotrophic factors are intimately involved in the development and survival of neurons thereby supporting their candidacy as a therapeutic option for ALS. MNs are able to bind, internalize, and retrogradely transport growth factors from muscle in a receptor-dependent manner. Alternatively, injecting viral constructs encoding growth factors directly into the spinal cord avoids the need for retrograde transport of the protein from the muscle. Several growth factors such as GDNF, IGF-I, vascular endothelial growth factor (VEGF), ciliary neurotrophic factor (CNTF), and brain-derived growth factor (BDNF) have been evaluated in experimental models of ALS (for review see [49, 50]). In nearly all cases, these factors have had positive effects on both MN survival and function in SOD1^G93A^ rodents [51–54]. 

GDNF is important in the branching of neurons at the NMJ and modulating synaptic plasticity [55]. The enhanced expression of GDNF in the muscle of the SOD1^G93A^ mice delays disease onset, improves locomotor performance, and increases lifespan [51–53, 56–58]. However, delivering GDNF directly to the MNs within the spinal cord had only modest effects on the survival of facial MNs and no effect on lumbar MN survival or function. This was observed even though high levels of GDNF were expressed directly around dying MNs [59]. In support of this study, another report used promoter-driven transgenic mice to overexpress GDNF locally in either the muscle or spinal cord of SOD1^G93A^ animals. GDNF expression in the muscle was able to slow disease progression and onset, but expression in the spinal cord had no effect [60].

In previous years, we demonstrated that intramuscular GDNF delivery using stem cells helps preserve NMJs (Figure 2(c)) [61]. Human mesenchymal stem cells (hMSC) were genetically modified to release GDNF (hMSC-GDNF) and were transplanted into the limb muscles of presymptomatic SOD1^G93A^ rats. These cells survived, released GDNF, and significantly affected innervation of NMJs in the transplanted muscle at 6 weeks post surgery. hMSC-GDNF transplanted rats also survived ~18 days longer than their control littermates when animals were kept until endpoint [61]. 

IGF-I has been known to play a key role in MN survival, axonal growth, and the maintenance of synaptic connections [62, 63]. This trophic factor is involved in muscle and nerve tissue anabolism and thus induces muscle hypertrophy and promotes neural survival. After intramuscular treatment with adeno-associated virus expressing IGF-I, it was shown that IGF-I can be retrogradely transported from muscle to the spinal cord and led to MN protection in the SOD1^G93A^ mice [52]. This effect was further increased when physical exercise was associated with treatment [64]. Another study reported that muscle-restricted expression of IGF-I isoforms maintained muscle integrity, stabilized neuromuscular junctions, enhanced MN survival, delayed the onset of disease, and slowed disease progression in the SOD1^G93A^ mice [65]. These studies reappraised the potential role of the skeletal muscle and IGF-I signaling as a target for treatment in ALS patients. 

VEGF is another trophic factor that contributes to the pathogenesis of ALS and possibly applies to muscle-target treatments. In SOD1^G93A^ mice, increased expression of VEGF by intramuscular viral injections prolongs their survival and enhances motor performance [7, 53]. Also, intracerebroventricular administration of VEGF in a rat model of ALS enhanced MN survival, while an intraperitoneal injection of VEGF led to the preservation of NMJs [66]. 

Despite the promising effects in preclinical studies, several growth factors, including BDNF, CNTF, and IGF-I, did not yield positive results in clinical trials for ALS patients [67]. However, the failure of these trials may be attributed to factors such as inappropriate delivery routes and doses which were validated in preclinical trials and may have affected the pharmacological concentration of growth factors in target tissues [68]. Therefore, the therapeutic benefits of these growth factors may need to be tested using the direct delivery into skeletal muscle.

NMJ degeneration may also be alleviated by controlling abnormally elevated energy metabolism which occurs in muscle. It has been suggested that hypermetabolism in skeletal muscle drives a chronic energy deficit in SOD1^G93A^ mice which precedes amyotrophy and muscle denervation. SOD1^G93A^ mice show a body weight deficit compared to wild type mice [33]. This body weight deficit was not due to decreased food intake, but rather to an increase in the basal metabolic rate. Energy metabolism, especially lipid metabolism, was strikingly altered in these animals. Furthermore, gene expression changes and increased muscle glucose uptake implicated the muscle as a site of excessive nutrient consumption in SOD1^G93A^ mice. Interestingly, a high-fat diet used to increase energy levels was enough to prolong the life of SOD1^G93A^ rats and reduce muscle denervation, although this strategy might not work well for human ALS patients due to insulin resistance [37, 69]. 

If an altered metabolic rate in skeletal muscle is critical for NMJ degeneration, exercise would also be expected to benefit ALS patients. Recent studies using ALS mouse models have reported a life span increase in exercised animals [70, 71]. Therapeutic exercise is also feasible, tolerated, and safe for patients with ALS [72, 73]. Clinical trials of ALS patients have suggested that regular physical exercise may be neuroprotective, ameliorate symptoms, and improve functionality [74]. Interestingly, synergistic effects of IGF-I gene delivery and exercise have profound effects on survival function [64]. Therefore, it is possible that combining exercise and stem-cell- or viral-based growth factor delivery may provide a more powerful therapy.

## 8. Conclusion

ALS is emerging as a “multisystemic” disease in which structural, physiological, and metabolic alterations occur in different tissues and cell types such as MNs, glia, and muscle tissues. The degenerating processes may act synergistically to induce and exacerbate the disease. Recent studies have provided evidence supporting a “dying-back” hypothesis in which distal NMJ degeneration precedes proximal neuronal cell death. It has been proposed that NMJ degeneration is not initially noticeable due to reinnervation processes by remaining axons of the muscle fibers as part of a compensatory mechanism. Eventually, however this process cannot keep up with the disease progression and muscle weakness is observed. Growth factor delivery targeting the skeletal muscle has provided significant results in protecting NMJ innervations, increasing MN survival, and prolonging the survival period of rodent models of ALS. On the other hand, treatments to rescue MNs according to a “dying-forward” model of MN pathology in ALS have shown only limited success in SOD1^G93A^ transgenic rodents as well as humans. Due to the accessibility of muscle tissue, it is much easier to directly deliver growth factors in muscle than in other tissues such as the spinal cord. Perhaps the most powerful approach will be to target both the spinal cord (i.e., cell body) and muscle (i.e., nerve terminals of MNs).
